# Cumulative Impacts and COVID-19: Implications for Low-Income, Minoritized, and Health-Compromised Communities in King County, WA

**DOI:** 10.1007/s40615-021-01063-y

**Published:** 2021-06-14

**Authors:** Carolyn Ingram, Esther Min, Edmund Seto, BJ Cummings, Stephanie Farquhar

**Affiliations:** 1 School of Public Health, Physiotherapy, and Sports Science, University College Dublin, Belfield, Dublin 4, Ireland; 2 ISPED (Bordeaux School of Public Health) , University of Bordeaux , Bordeaux, France; 3 Department of Environmental & Occupational Health Sciences, University of Washington, Seattle, WA USA; 4 Department of Health Services, University of Washington, Seattle, WA USA

**Keywords:** COVID-19, Racial disparities, Cumulative impacts, Environmental health disparities, Social determinants of health, Environmental exposures

## Abstract

Few studies have assessed how the intersection of social determinants of health and environmental hazards contributes to racial disparities in COVID-19. The aim of our study was to compare COVID-19 disparities in testing and positivity to cumulative environmental health impacts, and to assess how unique social and environmental determinants of health relate to COVID-19 positivity in Seattle, King County, WA, at the census tract level. Publicly available data (n = 397 census tracts) were obtained from Public Health–Seattle & King County, 2018 ACS 5-year estimates, and the Washington Tracking Network. COVID-19 testing and positive case rates as of July 12, 2020, were mapped and compared to Washington State Environmental Health Disparities (EHD) Map cumulative impact rankings. We calculated odds ratios from a series of univariable and multivariable logistic regression analyses using cumulative impact rankings, and community-level socioeconomic, health, and environmental factors as predictors and having ≥ 10% or < 10% census tract positivity as the binary outcome variable. We found a remarkable overlap between Washington EHD cumulative impact rankings and COVID-19 positivity in King County. Census tracts with ≥ 10 % COVID-19 positivity had significantly lower COVID-19 testing rates and higher proportions of people of color and faced a combination of low socioeconomic status–related outcomes, poor community health outcomes, and significantly higher concentrations of fine particulate matter (PM_2.5_). King County communities experiencing high rates of COVID-19 face a disproportionate cumulative burden of environmental and social inequities. Cumulative environmental health impacts should therefore systematically be considered when assessing for risk of exposure to and health complications resulting from COVID-19.

**Supplementary Information:**

The online version contains supplementary material available at 10.1007/s40615-021-01063-y.

## Introduction

Racial disparities in COVID-19 have been widely reported in the USA, where communities of color experience a disproportionate burden of infection and mortality [1]. Despite research demonstrating that neighborhood differences in health outcomes persist after adjustment for individual risk factors [2], few studies have assessed vulnerability to COVID-19 at the community level. Explanations have often focused on individual-level risk factors such as preexisting health conditions. People of color are at increased risk for comorbidities for severe COVID-19 like cardiovascular disease and diabetes [3] and may experience chronic and toxic stress brought on by racial discrimination that weakens the immune system [4]. Yet beyond individual-level risk factors, vulnerability in health tends to be concentrated in areas where social determinants of health (limited educational attainment, low SES, unemployment, discrimination) and built environmental inequities (limited access to health care, air quality) intersect and contribute to downstream adverse health outcomes [5, 6].

Community-level analysis, which relies on data aggregated by geographic boundaries such as census tracts, allows for the consideration of social and environmental determinants of health that may be contributing to COVID-19 disparities independently of individual-level attributes. The mechanisms by which community characteristics lead to racial health disparities are complex; however, structural and place-based factors such as systemic racism, residential racial segregation, concentrated poverty, access to healthcare, and proximity to environmental hazards are widely associated with adverse health outcomes [6–9]. Ongoing residential segregation of communities of color systematically shapes healthcare access, utilization, and quality at the community level [9]. By determining access to education and employment opportunities, it is a primary cause of racial differences in socioeconomic status [8]. Community socioeconomic characteristics in turn shape health-related behaviors and conditions such as health risk behaviors, overcrowding, and increased levels of environmental pollutants [6, 10, 11], which shape health outcomes [12, 13]. The result is a history of uneven spatial distribution, along racial lines, of infectious diseases [14]. Evaluating community differences in COVID-19 is therefore critical for highlighting place-based risks and resource deficits such as lack of preventive care services that may be contributing to uneven distribution of the epidemic [15].

Studies that have assessed racial disparities in COVID-19 at the community level have focused on social determinants of health like economic stability, education, housing conditions, and access to testing [16–19]. One assessment of US urban counties found that poorer areas with higher proportions of people of color experienced an excess burden of COVID-19 infection and death [16]. Another study from Massachusetts found that, along with higher proportions of Latino and Black residents, the proportion of foreign-born non-citizens, mean household size, and percentage of food service workers in a community were all positively associated with higher rates of COVID-19 [17]. In Louisiana, census tracts experiencing higher levels of area-based deprivation (a composite score based on measures of poverty, education, employment, and housing conditions) have higher rates of COVID-19 [18]; and in New York City, although residents of predominantly white zip codes are more likely to get tested for COVID-19, they are less likely to be positive [19]. These preliminary findings underline the importance of community-level analysis for identifying upstream determinants of COVID-19 and redirecting prevention resources to vulnerable populations.

Yet community-level research on COVID-19 disparities has rarely accounted for exposure to air pollution. Social inequities including concentrated poverty [20], higher rates of chronic health conditions [6], and lower levels of educational attainment [21] have all been linked to increased susceptibility to environmental health hazards. The relationship between environmental exposures and respiratory conditions found to fuel COVID-19 complications has been widely documented [22]. Emerging literature from China, California, and Italy indicates that areas with higher concentrations of ambient air toxics like fine particulate matter (PM_2.5_) and nitrogen dioxide (NO_2_) experience increased rates of COVID-19 [23–25]. Because communities of color in the USA reside in areas with worse air quality [26], these findings imply that exposure to ambient air toxics, especially when combined with social inequities, exacerbates racial disparities in SARS-CoV-2 infection and severity. Preliminary research confirms this, revealing a positive association between higher historical PM_2.5_ exposures and US county–level COVID-19 mortality rates after accounting for area-level confounders [27].

Cumulative environmental health impact analysis is a method for evaluating community susceptibility to environmental health hazards that considers both social and environmental determinants of health. The method assigns composite cumulative impact rankings based on pollution burden and population vulnerability across geographic bounds. These rankings indicate the potential a community has for combined and interactive exposure to environmental hazards and socioeconomic stressors that contribute to environmental health disparities [28]. Previously used in California and Washington State [26, 29], the cumulative impact approach can encourage the incorporation of equity and environmental justice goals into disease prevention response. The fact that existing community-level research has focused on social but not cumulative environmental determinants of COVID-19 may be contributing to why disease control resources are missing vulnerable populations in urban centers around the country [19].

In Seattle, King County, WA, the site of the USA’s first COVID-19 outbreak, local agencies led by Public Health–Seattle & King County have been proactive in mobilizing increased testing facilities in highly positive communities and high-volume vaccination programs and sites. Nevertheless, data indicate that communities of color in King County remain disproportionately impacted by the epidemic [30, 31]. Cumulative environmental health impact analysis, not yet applied to the regional COVID-19 context, can provide critical insight on ways to help close this health gap.

The goal of this study is to compare census tract–level COVID-19 disparities in testing and positivity to cumulative environmental health impacts in Seattle, King County, WA. We then aim to assess how unique social and environmental determinants of health relate to COVID-19 positivity at the census tract level. We hypothesize that in King County, communities facing the greatest cumulative impacts are disproportionately burdened by COVID-19 and in need of upscaled prevention resources.

## Methods

### Measures

#### Cumulative COVID-19 Data on Testing and Positivity

On July 12, 2020, during the summer peak of infection in King County, we obtained the cumulative number and rate of COVID-19 tests, positive cases, hospitalizations, and deaths recorded per King County census tract from the Public Health–Seattle & King County daily COVID-19 summary dashboard [30]. Public Health–Seattle & King County assigns COVID-19 tests and cases to an individual’s street address, counting residents with more than one test and/or positive test result only one time. Those with no street address, for example, homeless populations, are not included in census tract–level COVID-19 totals. COVID-19 positivity was calculated by dividing the total number of unique census tract residents with a positive PCR laboratory result reported to the Washington State Department of Health (DOH) by the total number of unique census tract residents with a reported PCR laboratory result. Tests for which results were pending as of July 12 were excluded from the denominator. We chose to measure COVID-19 disparities using testing rates and positivity in order to account for both access to and use of prevention resources (testing), and vulnerability to community spread (positivity). We did not measure COVID-19 disparities using hospitalization and/or death rates as the geospatial distribution of COVID-19 morbidity and mortality was largely linked to outbreaks in long-term care facilities as of July 2020.

#### Washington Environmental Health Disparities Map Cumulative Impact Rankings

We downloaded census tract–level cumulative impact rankings, environmental exposures rankings, environmental effects rankings, sensitive populations rankings, and socioeconomic factors rankings from the Washington Environmental Health Disparities (EHD) Map [32]. The EHD Map is an interactive tool that compares communities across Washington State for environmental health disparities using 19 census tract–level indicators divided into four categories:

- *Environmental exposures* (NO_x_-diesel emissions; ozone concentration; PM_2.5_ concentration; populations near heavy traffic roadways; toxic releases from facilities)
- *Environmental effects* (lead risk and exposure, proximity to hazardous waste, proximity to Superfund sites, proximity to facilities with highly toxic substances, wastewater discharge)
- *Sensitive populations* (cardiovascular disease, low birth weight)
- *Socioeconomic factors* (people of color, poverty, education level, housing and transportation expense, linguistic isolation, unemployment)

Washington State census tracts are assigned a relative, decile score for each of the 19 indicators based on rank-order of the raw values, as detailed by Min et al. [29]. Census tracts receive a ranking from 1 to 10 in the four listed categories by taking the average decile score of individual indicators. Composite, cumulative impact scores are then calculated and ranked according to deciles (each decile represents 10% of Washington State’s 1463 census tracts):

$$ Cumulative\ Impact\ Score=\frac{Environmental\ Exposures\ Rank+0.5\ast Environmental\ Effects\ Rank}{2}\ast \frac{Sensitive\ Population\ Rank+ SES\  Factors\ Rank}{2} $$

The composite, relative rankings allow for assessment of the cumulative impact of multiple indicators across communities. Communities with a cumulative impact ranking of 9 or 10 are considered highly impacted by existing environmental health disparities and thus at risk for adverse health outcomes. Because Washington EHD cumulative impact rankings were developed through a community-engaged process that explored upstream exposures and vulnerability factors that impact environmental health disparities broadly, they can serve as a good preliminary indicator of community vulnerability to COVID-19.

#### Community-Level Social and Environmental Determinants of Health

**Fig. 1:**
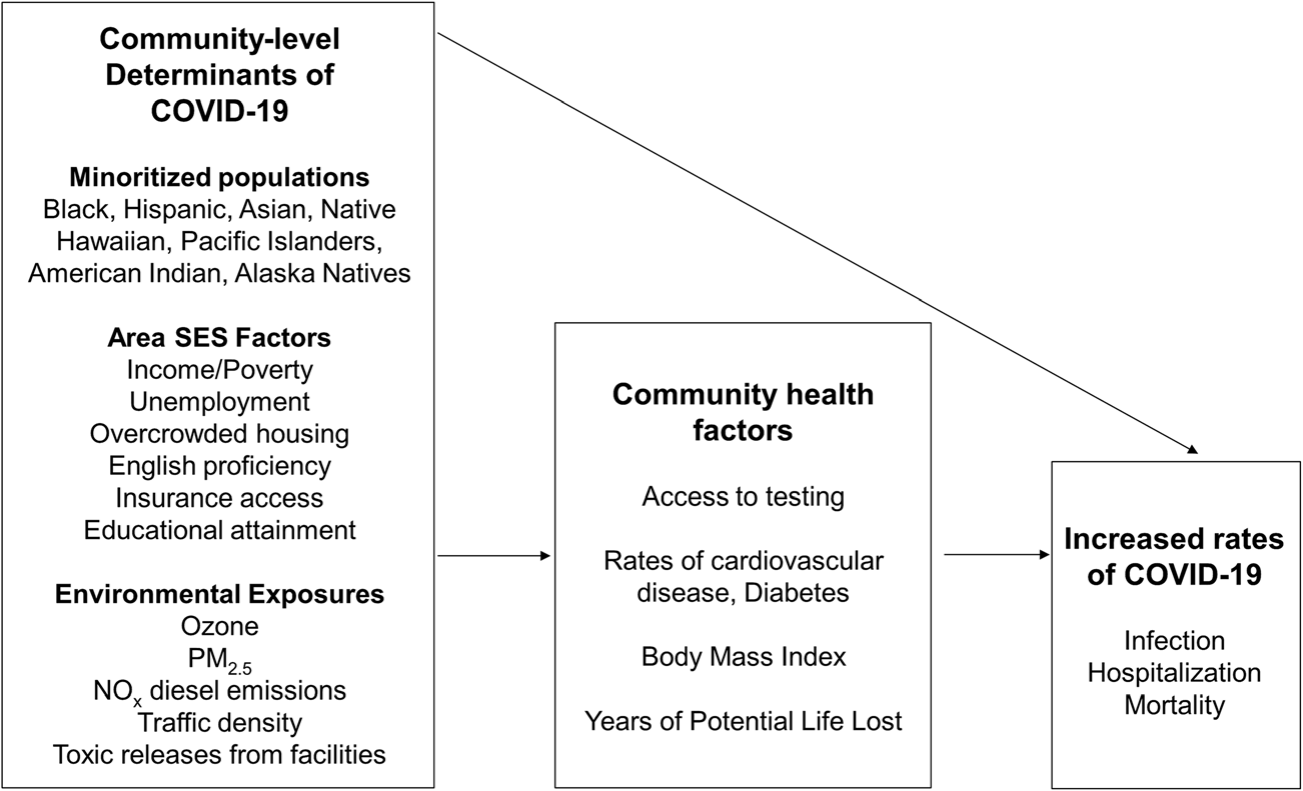
A conceptual framework for the association between neighborhood-level social and environmental determinants of health and community vulnerability to COVID-19: Seattle, King County, WA, 2020. *Note: Figure shows association between community determinants of health (minoritized populations, socioeconomic and environmental factors) and COVID-19. Community health factors are identified risk factors for SARS-CoV-2 infection and/or severity of infection and are potential mediators on the pathway between race, SES, air pollution, and COVID-19 disparities. Note that factors relating to healthcare access, essential workforce, and respiratory health outcomes were not publicly available at the census tract level in Washington State and could not be included in this analysis*

As Washington EHD cumulative impact rankings are not intended to diagnose specific community health issues [29], additional analysis is needed to identify how COVID-19 disparities are affiliated with unique community-level social and environmental risk factors. This knowledge is critical for suggesting targeted policy interventions to address racial disparities in COVID-19 and at the outset of future epidemics. To assess how specific inequities relate to COVID-19 positivity in King County, we developed a conceptual model of the association between community-level social and environmental determinants of health and COVID-19 (Fig. 1), guided by Lowcock et al. [33]. Included census tract–level indicators met two criteria: being a known or suspected risk factor for SARS-CoV-2 infection or severity of infection and being publicly available at the census tract level. While there is some overlap between indicators included in our conceptual framework and indicators included in Washington EHD cumulative impact rankings, these are two separate exposure sets. Our list of community-level variables was not meant to be exhaustive but to provide a preliminary understanding of how race, SES, community health, and environmental quality all relate to COVID-19 in King County census tracts.

We used the 2014–2018 American Community Survey (ACS) 5-year estimates for census tract–level data on race (ACS Table: DP05) and percentage living below 185% federal poverty level (FPL) (e.g., an annual household income of ≤ $48,000 for a family of four [34]); ACS Table: S1701). The race and ethnicity groups included in this study were Black, Hispanic, Asian, Native Hawaiian or Other Pacific Islander, American Indian, or Alaska Native. Groups were mutually exclusive, and being of Hispanic/Latino origin was counted as a race in order to be consistent with Public Health–Seattle & King County’s reporting of COVID-19 by race. A person of color was defined as anyone who did not identify exclusively as non-Hispanic white. We downloaded census tract–level socioeconomic, health-related, and environmental indicators from the Washington Tracking Network [35].

To account for COVID-19 testing access, we recorded the locations of testing facilities on July 5, 2020, from King County’s Open Access Testing Locations Tool. To account for facility-based outbreaks, on July 24, 2020, we recorded the locations of long-term care facilities with 5 or more deaths due to COVID-19 from the Public Health–Seattle & King County Long Term Care Data Dashboard.

### Analysis

Our assessment of cumulative impacts and COVID-19 was conducted in multiple stages. We first mapped rates of COVID-19 tests and positive cases per 1000 census tract residents and COVID-19 positivity as of July 12, 2020, according to quintiles and conducted side-by-side visual comparisons with the distribution of Washington EHD cumulative impact rankings.

We then evaluated how Washington EHD cumulative impact rankings and individual census tract–level factors related to COVID-19 disparities by dividing census tracts into two groups: high positivity (census tracts with greater than or equal to 10% positivity) and referent (census tracts with less than 10% positivity). The World Health Organization (WHO) uses 10% positivity as their testing capacity target. If more than 10% of test results are positive in a community, active cases are likely to be missed and case numbers will continue rising [36]. To assess the associations between COVID-19 positivity and our first exposure set (Washington EHD cumulative impact rankings and subgroup rankings), odds ratios (OR) generated from logistic regression models were used to estimate the likelihood of having ≥ 10% vs < 10% census tract positivity. The first, univariable model assessed the association between COVID-19 positivity and overall Washington EHD cumulative impact rankings. The second, multivariable model assessed the association with COVID-19 positivity and individual subgroup rankings for environmental exposures, environmental effects, sensitive populations, and socioeconomic factors.

Using our second exposure set (social and environmental factors from our conceptual framework), we summarized independent community-level indicators in high positivity and referent census tracts (mean, median, standard deviation). We calculated odds ratios with a series of univariable logistic regression using independent community-level factors as predictors and having ≥ 10% or < 10% census tract positivity as the binary outcome variable. To account for multiple comparisons, a Bonferroni correction was applied, and the significance threshold was set at P <0.01. We conducted additional multivariable logistic regression analyses testing associations between the outcome variable and SES, community health, and environmental factors, separately. Because of potential collinearity among the independent variables, Pearson correlations were used to identify highly correlated variables (> 0.70). Since we were interested in how each community-level characteristic related to COVID-19 positivity, we chose not to combine SES, health, and environmental variables in the same model to avoid having highly correlated variables in the same model. The significance threshold for all adjusted odds ratios (AOR) was set at P < 0.05. We mapped the locations of no-insurance-required testing facilities in relation to ≥ 10 % positive census tracts in order to assess proximity to free testing. Finally, we undertook a sensitivity analysis of the effect of restricting analyses to census tracts with no facility-based outbreaks, and of using other internationally recognized COVID-19 positivity thresholds (5%, 15%). All statistical analysis was completed using R version 4.0.2 (R Foundation for Statistical Computing, Vienna, Austria). Maps were created with the leaflet 2.0.3 package.

## Results

**Fig. 2:**
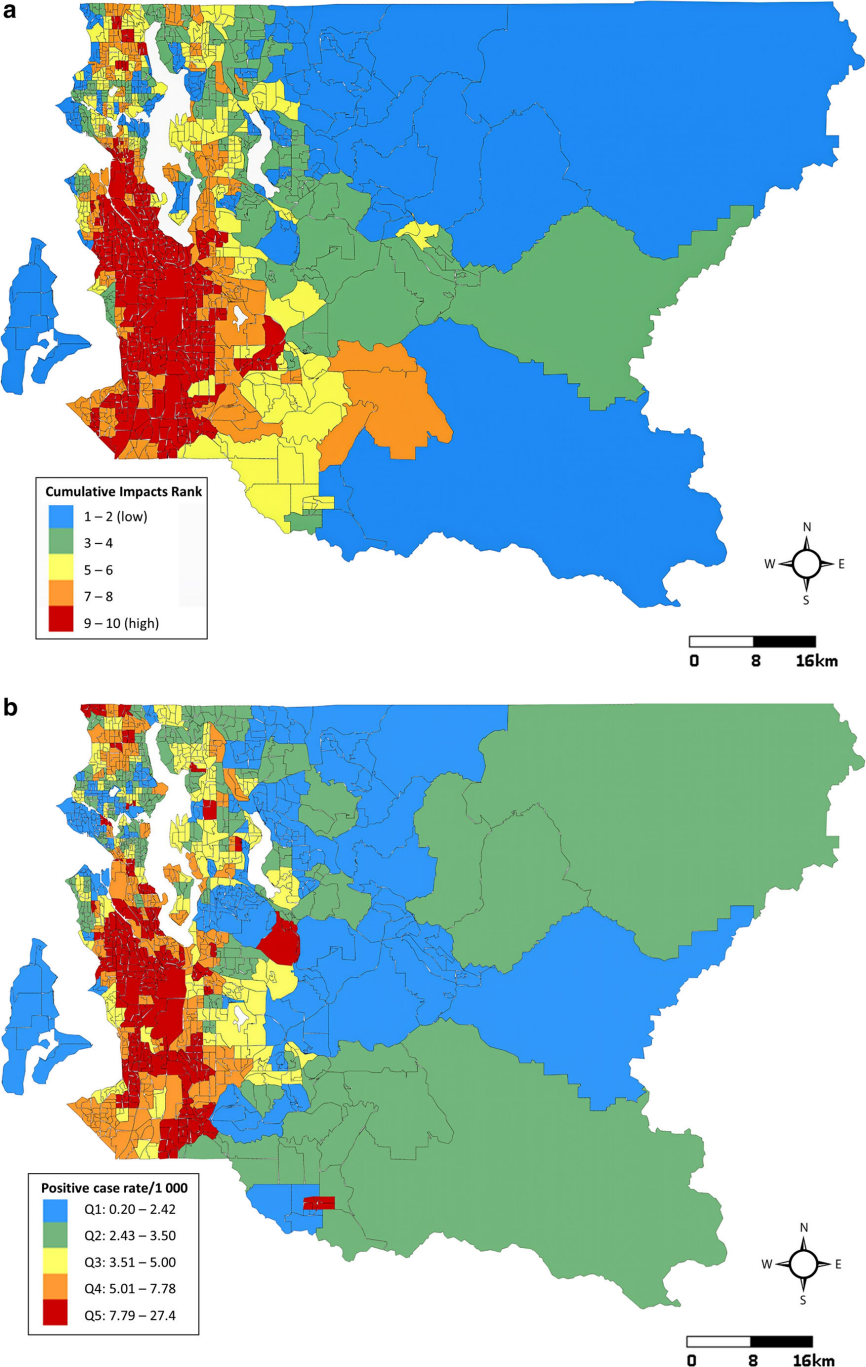

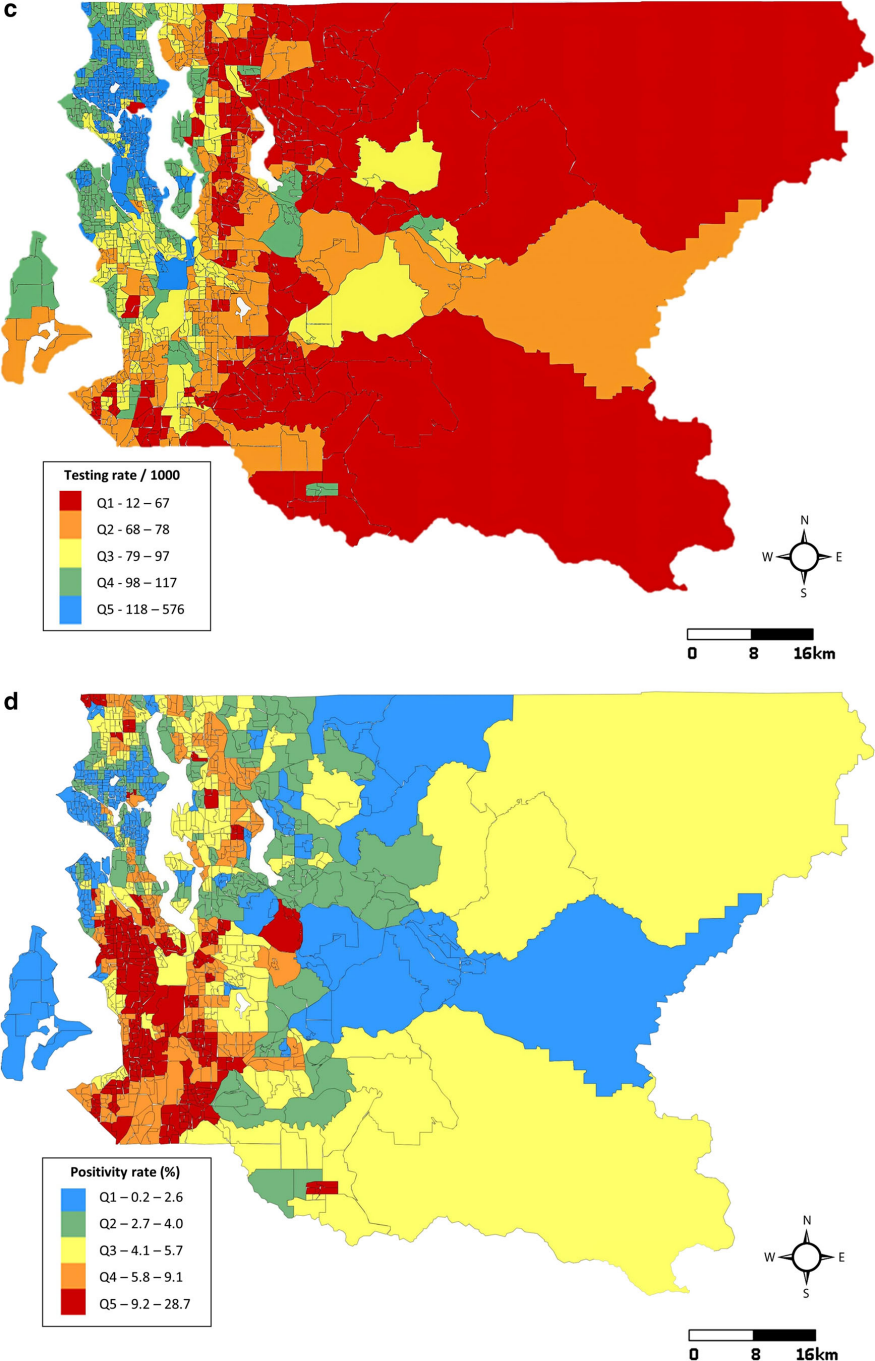
Comparison of Washington Environmental Health Disparities Map cumulative impact rankings (**a**), cumulative positive COVID-19 case rates per 1000 residents (**b**), cumulative COVID-19 testing rates per 1000 residents (**c**) and cumulative COVID-19 positivity (%) (**d**) in 397 census tracts: Seattle, King County, WA, July 12, 2020

In Seattle and King County, WA, 397 census tracts have an average of 5449 residents each (range: 1115–14,540), accounting for 2.16 million residents in total. As of July 12, 2020, the average cumulative COVID-19 testing rate across census tracts was 94.8 tests per 1000 residents (SD = 38.3). The average cumulative positive case rate was 5.3 cases per 1000 residents (SD = 3.6) and overall county positivity was 5.6%. Census tract positive case rates, positivity, and testing rates as of July 12 and cumulative impact rankings from Washington’s EHD mapping tool are displayed in Fig. 2. At a glance, areas experiencing high rates of COVID-19 were disproportionately burdened by cumulative environmental health impacts. Census tracts with the highest rates of positive cases were clustered in the southwestern part of the county where environmental health disparities were highest. These same areas had lower testing rates than the northwestern part of the county, where positive case rates and environmental health disparities were lower.

**Fig. 3:**
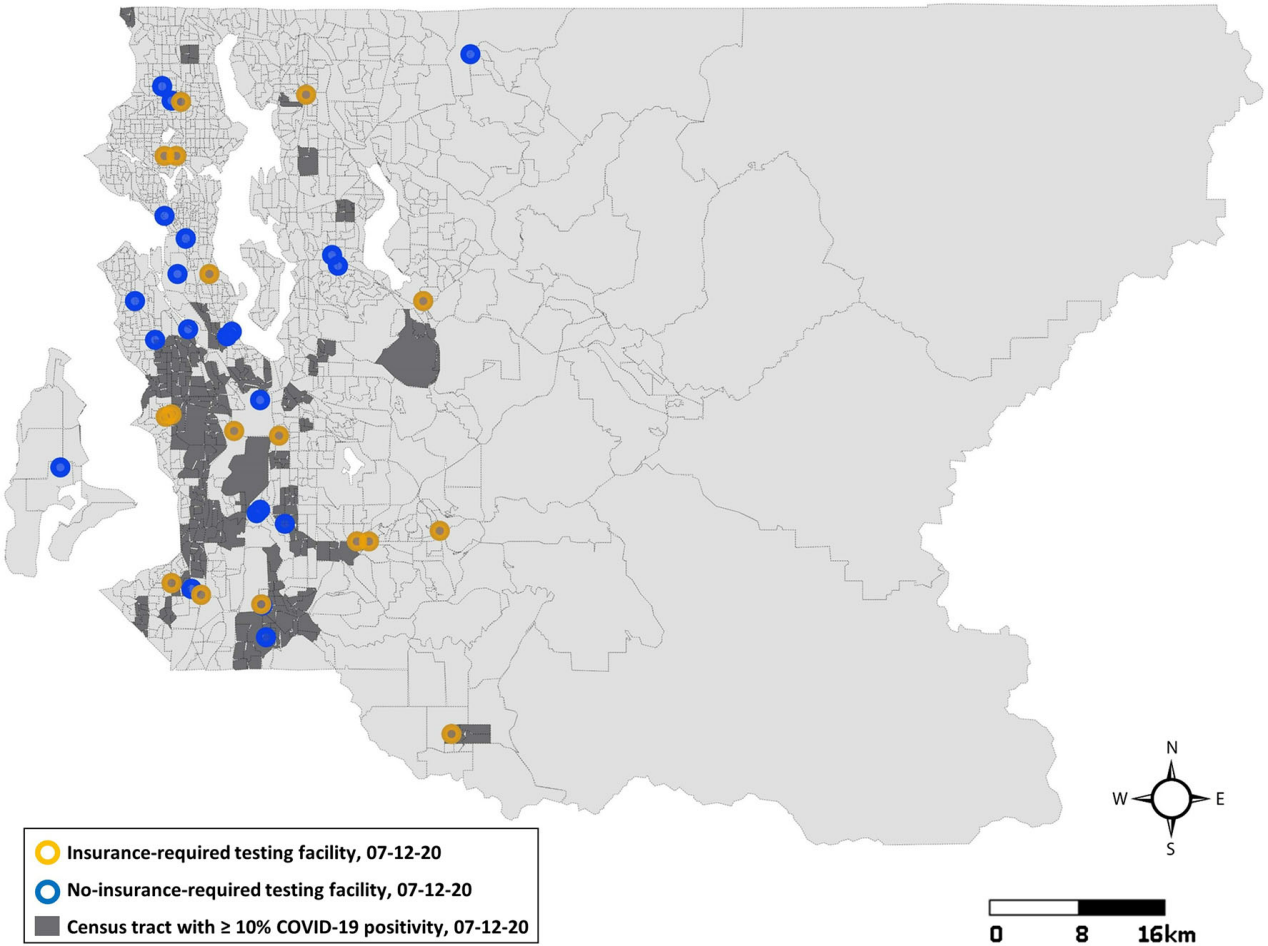
Locations of testing facilities in proximity to census tracts with ≥ 10% positive test results: Seattle, King County, WA, July 12, 2020

As of July 12, 2020, 64 of King County’s 397 census tracts had high positivity (≥ 10%). Residents of these 64 tracts accounted for 16.0% (346,613/2,163,257) of King County’s population. In high positivity tracts, 34.1% of residents were living under 185% FPL, compared to 19.4% in all King County. People of color represented 58.1% of the population, compared to 39.6% in all King County. Ten of the 64 tracts were the site of facility-based outbreaks (Supplemental Figure 1). High positivity census tracts had significantly higher positive case rates (OR = 2.2; 99% CI = 1.7, 2.8), hospitalization rates (OR = 4.91; 99% CI = 2.66, 9.05), and mortality rates (OR = 2.78; 99% CI = 1.40, 5.52), but received only 81 tests per 1000 residents on average, compared to 97 in referent tracts (OR = 0.98; 99% CI = 0.97, 1.0). Figure 3 shows the locations of high positivity census tracts and no-insurance-required testing facilities as of July 12 and reveals a gap in free testing in the western part of southwest King County.

Table 1 shows summary statistics and logistic regression results for the association between high COVID-19 positivity and tract-level Washington EHD Map cumulative impacts and between high COVID-19 positivity and Washington EHD cumulative impact subgroups. High positivity census tracts had an average cumulative impact ranking of 9.0 (SD = 1.9) compared to 5.9 (SD = 2.7) in referent tracts (OR = 2.0; 95% CI = 1.6, 2.4). Only 11 of the 64 high positivity tracts had cumulative impact rankings below the highly impacted level (ranks < 9), and six of these were the site of facility-based outbreaks. While SES factors, sensitive populations, environmental exposures, and environmental effects were all associated with high COVID-19 positivity, only SES remained significantly associated when adjusted for other subgroup rankings (OR = 1.9; 95% CI = 1.5, 2.3).

**Table 1:** Summary statistics and logistic regression results for census tract COVID-19 positivity (≥ 10 % vs < 10%) based on Washington Environmental Health Disparities Map cumulative impact rankings and subgroup rankings: 397 census tracts, Seattle, King County, WA, July 12, 2020

	≥ 10 % CTs (n = 64)		< 10 % CTs (n = 333)			
	Mean	SD	Mean	SD	Crude OR (95% CI)	Adjusted OR (95% CI)
Model 1—Washington EHD cumulative impact rankings						
Cumulative impact rank^a^	9.01	1.91	5.88	2.71	1.98 (1.62, 2.42)**	
Model 2—Washington EHD subgroup rankings						
SES factors rank^b^	8.53	1.89	4.20	2.76	1.97 (1.66, 2.34)**	1.87 (1.53, 2.28)**
Sensitive populations rank^c^	7.02	2.33	4.60	2.72	1.39 (1.25, 1.56)**	1.10 (0.96, 1.26)
Environmental exposures rank^d^	8.81	1.78	7.19	2.06	1.70 (1.36, 2.08)**	1.16 (0.93, 1.44)
Environmental effects rank^e^	8.55	1.91	6.56	2.65	1.47 (1.27, 1.70)**	0.97 (0.81, 1.16)

*p < 0.05; **p < 0.01

^a^Relative, composite measure of pollution burden (environmental effects + exposures) × population vulnerability (SES factors + sensitive populations) for all Washington State census tracts

^b^Average of Washington State census tract relative decile rankings for people of color, poverty, education level, housing and transportation expense, linguistic isolation, unemployment

^c^Average of Washington State census tract relative decile rankings for cardiovascular disease, low birth weight

^d^Average of Washington State census tract relative decile rankings for NOx-diesel emissions; ozone concentration; PM_2.5_ concentration; populations near heavy traffic roadways; toxic releases from facilities

^e^Average of Washington State census tract relative decile rankings lead risk and exposure, proximity to hazardous waste, proximity to Superfund sites, proximity to facilities with highly toxic substances, wastewater discharge

Unique social and environmental determinants of health are described according to COVID-19 positivity group in Table 2 along with odds ratios generated from univariable logistic regression. Individual community-level factors significantly associated with high positivity included the proportion of people of color in a census tract (OR = 1.08; 99% CI = 1.05, 1.10), in particular, the proportion of Hispanic (OR = 1.23; 99% CI = 1.16, 1.31), Black (OR = 1.12; 99% CI = 1.07, 1.17), and Native Hawaiian/Pacific Islander residents (OR = 1.5; 99% CI = 1.2, 1.8); and the proportion of residents living below 185 % FPL (OR = 1.09; 99% CI = 1.06, 1.13), residents over 25 years old with no high school diploma (OR = 1.10; 99% CI = 1.06, 1.13), uninsured (OR = 1.11; 99% CI = 1.05, 1.16) and unemployed residents (OR = 1.17; 99% CI = 1.03, 1.32), residents who spoke English less than very well (OR = 1.08; 99% CI = 1.04, 1.12), and those who lived in residences with more than one occupant per room (OR = 1.47; 99% CI = 1.29, 1.68). Rates of cardiovascular (OR = 4.7; 99% CI = 2.6, 8.6) and diabetes-related mortality per 1 000 (OR = 15.6; 99% CI = 5.4, 45.2) were also significantly associated with high positivity, as was body mass index (BMI) (OR = 10.82; 99% CI = 2.6, 8.6). For every 1000 residents living in a high positivity census tract, 18.4 more years of potential life (YPLL) were lost on average (OR = 1.08; 99% CI = 1.04, 1.11). Concerning air pollution, the 3-year mean PM_2.5_ concentration (OR = 5.5; 99% CI = 2.9, 10.3) and the percentage of the population living near heavy traffic roadways (OR = 1.02; 99% CI = 1, 1.03) were significantly higher in census tracts with high positivity than in referent tracts. Concentrations of ozone, nitrogen oxides (NOx), and toxic releases from facilities did not vary significantly (OR = 0.98; 99% CI = 0.85, 1.11; OR = 0.98; 99% CI = 0.95, 1.01; and OR = 1; 99% CI = 1, 1, respectively).

**Table 2:** Description and univariable logistic regression results for community-level characteristics of 397 census tracts according to COVID-19 positivity group: Seattle, King County, WA, July 12, 2020

	≥ 10 % census tracts (n = 64) Population: 346,613			< 10 % census tracts (n = 333) Population: 1,816,644			
	Mean	Median	SD	Mean	Median	SD	OR (99% CI) ^q^
COVID-19 outcomes							
Test rate 07-12^a^	82.5	81.1	17.6	97.2	93.6	40.6	0.98 (0.97, 1)
Positive case rate 07-12 ^a^	11.0	10.3	3.44	4.16	3.70	2.26	2.18 (1.72, 2.76)
Hospitalization rate 07-12 ^a^	1.66	1.20	2.01	0.56	0.40	0.54	4.91 (2.66, 9.05)**
Mortality rate 07-12 ^a^	0.69	0.20	1.48	0.19	0.00	0.33	2.78 (1.40, 5.52)^+^**
Race							
People of color (%) ^b^	57.0	57.2	15.0	35.4	32.7	16.7	1.08 (1.05, 1.10)**
Hispanic (%)^c^	20.70	19.22	9.16	8.98	7.42	5.16	1.23 (1.16, 1.31)**
Black (%)	12.41	10.80	8.78	4.89	2.20	6.27	1.12 (1.07, 1.17)**
Asian (%)	16.72	14.45	10.77	16.54	14.0	11.65	1.00 (0.97, 1.03)
American Indian/Alaska Native (%)	0.68	0.30	0.93	0.47	0.20	1.14	1.13 (0.87, 1.46)
Native Hawaiian/other Pacific Islander (%)	2.25	1.15	2.84	0.52	0.00	1.40	1.47 (1.23, 1.77)**
Socioeconomic factors							
< 185 % federal poverty level (%)^d^	34.25	34.20	10.65	17.76	14.36	11.99	1.09 (1.06, 1.13)**
No high school diploma (%)^e^	13.63	12.77	7.78	6.57	4.25	6.34	1.13 (1.07, 1.18)**
Uninsured (%)^f^	13.81	13.38	6.83	8.76	7.18	6.22	1.11 (1.05, 1.16)**
Not fluent in English (%)^g^	15.87	14.12	8.60	9.48	7.00	8.14	1.08 (1.04, 1.12)**
Unemployed (%)^h^	6.11	5.94	2.82	4.87	4.36	2.56	1.17 (1.03, 1.32)*
Overcrowded housing (%)^i^	7.20	6.24	4.03	2.78	2.02	2.62	1.47 (1.29, 1.68)**
Community health factors							
Cardiovascular mortality rate^a^	2.43	2.29	0.79	1.72	1.64	0.57	4.68 (2.55, 8.61)**
Diabetes-related mortality rate^a^	0.98	0.89	0.46	0.58	0.51	0.32	15.61 (5.39, 45.24)**
Years of potential life lost^aj^	39.8	41.1	11.4	25.5	22.7	13.1	1.08 (1.04, 1.11)**
Body mass index^k^	26.4	26.5	0.68	25.2	25.2	0.76	10.82 (5.02, 23.33)
Environmental exposures							
PM_2.5_ concentration^l^	6.92	7.12	0.66	6.05	6.09	0.80	5.47 (2.92, 10.26)**
Ozone concentration^m^	50.5	49.8	2.29	50.7	49.8	2.85	0.98 (0.85, 1.11)
NO_x_ concentration^n^	12.9	13.5	5.04	17.9	12.3	18.5	0.98 (0.95, 1.01)
Population near heavy traffic roadway (%)^o^	23.6	20.3	22.5	14.62	1.23	21.8	1.02 (1, 1.03)*
Toxic releases from facilities^p^	19,711	13,321	18,722	19,338	9317	29,278	1 (1, 1)

*p <0.01; **p < 0.001

+Results no longer significant after exclusion of the 23 census tracts with identified facility-based outbreak (10/64 high positivity tracts, 13/333 referent tracts, p = 0.07)

^a^Rates per 1000 census tract population

^b^Refers to anyone who does not identify exclusively as non-Hispanic white. All race data from 2018 ACS 5-year estimates

^c^Hispanic as race. The following race categories are mutually exclusive: Hispanic, Black, Asian, AIAN, NHPI

^d^Percent of census tract residents living in households making less than 185% FPL (i.e., $48,000 for a family of four)

^e^Percent of census tract residents who have not received a high school diploma or GED by the age of 25. Data for socioeconomic, health, and environmental factors accessed via Washington Tracking Network (WTN)

^f^Percent of total civilian non-institutionalized population, ages 18 to 65 that do not have health insurance per census tract. Health insurance includes both private and public (e.g., Medicaid)

^g^Percent of census tract residents age 5+ speaking English less than very well

^h^Percent of census tract population 16 years and older that are in the labor force and registered as unemployed

^i^Percent of census tract residents living in housing where there is more than one person per room

^j^Premature mortality uses the age when a person died based on a life expectancy to age 65 and takes those years as the years of potential life lost

^k^Age-adjusted BMI for all census tract residents (Sum of resident heights/sum of age-adjusted resident weights). Three-year mean concentration of annual PM2.5 for 2009–2011 in micrograms per cubic meter (μg/m^3^). Maximum healthy concentration is 10 μg/m^3^, WHO Air Quality Guidelines 2005

^l^Three-year mean concentration of daily maximum 8-h rolling averaged ozone for 2009–2011 (μg/m^3^). Healthy guideline level is 100 μg/m^3^, WHO Air Quality Guidelines 2005

^m^NOx-diesel emissions in annual tons per square kilometer. Maximum healthy concentration is 100 tons/km2, EPA 2001

^n^Percentage of the population living exposed to busy roadways (within 300 m on either side)

^o^The toxicity-weighted concentrations of chemical releases to air from facility emissions and off-site incineration averaged over a three-year period

^p^Univariable logistic regression analysis. Significance threshold set at P < 0.01 after application of Bonferroni correction

Before assessing the association between groups of SES factors, community health factors, environmental factors, and COVID-19 positivity, Pearson correlations were computed between tract-level variables of the same group. English and education level were found to be highly correlated (0.796), as were BMI and diabetes-related mortality (0.70); thus, only the more upstream determinants (education level and BMI) were kept in the SES and community health multivariable models. The SES logistic regression model estimated associations between high COVID-19 positivity, area SES factors, and the percentage of people of color in a census tract (Table 3). Adjusted for other SES factors, overall poverty (AOR = 1.05; 95% CI = 1.02, 1.08), education levels (AOR = 1.05; 95% CI = 1.00, 1.10), and overcrowded housing (AOR = 1.25; 95% CI = 1.10, 1.42), remained significantly associated with high COVID-19 positivity, whereas the association between the percentage of people of color and COVID-19 positivity was attenuated (AOR = 1.01; 95% CI = 0.98, 1.03). The community health logistic regression model estimated associations between tract-level health factors and high COVID-19 positivity. Only BMI was significantly associated with high positivity after adjustment for cardiovascular mortality, YPLL, and access to medical insurance (AOR = 8.23; 95% CI = 4.00, 16.9). The last model, which examined the relationship between census tract positivity and environmental exposures, showed that higher concentrations of PM_2.5_ significantly increased the likelihood of high COVID-19 positivity after adjustment for other environmental exposures and population density (AOR = 3.7; 95% CI = 2.2, 6.4). NO_x_ concentration was significantly negatively associated with high positivity (AOR = 0.94; 95% CI = 0.89, 0.98). Neither ozone, toxic waste, nor the percentage of a census tract population living near heavy traffic roadways had a strong influence on COVID-19 positivity when adjusted for PM_2.5_, NO_x_, and population density.

**Table 3:** Multivariable logistic regression results for census tract COVID-19 positivity (≥ 10 % vs < 10%) based on SES factors, community health factors, and environmental exposures: 397 census tracts, Seattle, King County, WA, July 12, 2020

SES model		Community health model		Environmental exposures model	
	Adjusted OR (95% CI)		Adjusted OR (95% CI)		Adjusted OR (95% CI)
People of color (%)	1.01 (0.98, 1.03)	Cardiovascular mortality rate	1.27 (0.70, 2.29)	PM_2.5_	3.71 (2.16, 6.38)**
< 185 % federal poverty level (%)	1.05 (1.02, 1.08)**	YPLL	1.01 (0.97, 1.04)	Ozone	1.06 (0.90, 1.24)
No high school diploma (%)	1.05 (1.00, 1.10)*	BMI	8.23 (4.00, 16.9)**	NO_x_	0.94 (0.89, 0.98)*
Unemployed (%)	0.96 (0.85, 1.09)	Insurance (%)	1.02 (0.97, 1.07)	Population near heavy traffic (%)	1.02 (1.00, 1.04)
Overcrowded housing (%)	1.25 (1.10, 1.42)**			Toxic releases from facilities	1.00 (1.00, 1.00)
				Overcrowded housing (%)^+^	1.42 (1.25, 1.60)

*p <0.05; **p < 0.01

+The overcrowded housing indicator was included in the environmental exposures model to verify whether the correlation between air pollutants and COVID-19 infection was driven by high population density, as has been hypothesized elsewhere [37]

Though high positivity census tracts were home to nearly half of identified facility-based outbreaks (10/23), sensitivity analysis excluding all census tracts where facility-based outbreaks had occurred affected only one overall result: COVID-19 mortality rates no longer differed significantly between positivity groups during univariable regression analysis (OR = 2.41; 99% CI =0.7, 8.34). Further sensitivity analyses verified the appropriateness of the 10% positivity threshold, as other cut offs resulted in less meaningful comparisons between positivity groups (192/397 census tracts had ≥ 5% positivity on July 12; 17 had ≥ 15% positivity).

## Discussion

This study was the first to apply the Washington Environmental Health Disparities mapping tool in the context of the COVID-19 outbreak in Seattle, King County, WA. We found remarkable overlap between the Washington EHD Map and COVID-19 positivity in King County. Geospatial mapping and univariable logistic regression showed that census tracts with the highest cumulative impact rankings were facing a disproportionate burden of COVID-19 infection. These same communities were experiencing lower testing rates, indicating that highly impacted King County communities are in need of upscaled COVID-19 prevention resources. Of the subcategories of inequities contributing to Washington EHD cumulative impacts, multivariable logistic regression showed that SES and race were the greatest contributors to community-level COVID-19 disparities, highlighting a need for long-term structural interventions that address systemic racism and socioeconomic inequities in addition to short-term disease prevention and control solutions.

While Washington EHD cumulative impact rankings proved a rapid and effective tool for identifying community vulnerability to COVID-19, further analysis showed the value of tailoring cumulative impacts to the current epidemic. By assessing how unique community-level factors related to COVID-19 positivity, our study provides evidence of significant social and environmental inequities in communities facing high rates of COVID-19. Low socioeconomic status, existing health disparities, and poor air quality intersect resulting in that the communities most exposed to COVID-19 are the least equipped to combat its financial and health consequences. Logistic regression models point to where resources can be redirected to halt this cycle. For example, socioeconomic and health factors like crowded living conditions and BMI contribute to COVID-19 positivity independently of other racial, SES, and health disparities.

Our findings that highly COVID-19-positive census tracts had significantly higher proportions of Hispanic, Black, and low-income residents are consistent with other community-level studies [16, 17]. Multivariable analysis showed that the relationship between the percentage of people of color in a census tract and community COVID-19 positivity was attenuated after adjustment for socioeconomic inequities, underlining the role of SES as an intermediary between racial segregation and health disparities. We observed that overcrowded housing and levels of educational attainment were the SES-related factors most significantly associated with high census tract positivity. Although further research is needed to understand how exactly socioeconomic inequities are contributing to community differences in COVID-19, these findings point to several possibilities. For example, preventive measures like social distancing and self-isolation are likely impeded in south King County by housing and exposure through essential jobs, where frontline workers come into close contact with the public and may not be able to afford time off work if sick.

Residents in highly positive census tracts faced the added burden of higher BMI, higher rates of cardiovascular and diabetes-related mortality, shorter overall life expectancies, and, consequently, higher rates of COVID-19 hospitalizations. Multivariable logistic regression that accounted for multiple health inequities showed that BMI was the strongest underlying mediator on the pathway between race, SES, air pollution, and COVID-19. This is coherent with obesity (BMI > 30), which disproportionately impacts communities of color [38], being both an independent risk factor for COVID-19 and a risk factor for COVID-19 comorbidities like heart disease, lung disease, and diabetes [39]. Not only are severe outcomes of COVID-19 more likely in community members with underlying comorbidities, but the COVID-19 pandemic may impede their access to care. Reduced public transit can make it harder to attend medical appointments; fear of COVID-19 infection might prevent others from seeking healthcare at all. Virtual consultations are one solution; however, 67,000 King County households (7.5%) lack the necessary Internet access [40]. Encouragingly, local public health agencies have begun engaging with community advocates on ways to combat healthcare-related challenges [41]. With the epidemic ongoing, our study highlights a need for increased understanding of how to improve social and healthcare services for the county’s most vulnerable communities.

Similar to the results from New York City [19], we found that highly positive census tracts with high proportions of people of color experienced below average testing rates. This may be in part due to a gap in no-insurance-required testing facilities in the southwest part of King County as of mid-July. Nevertheless, some highly impacted communities near free testing services still had low testing rates, indicating that barriers other than proximity to facilities and/or expense may be keeping residents from getting tested. For minority populations, medical mistrust may be a reason not to seek out a COVID-19 test [42]. Dark moments in American history like the Tuskegee Syphilis Study continue to influence attitudes in the African American community towards biomedicine, as do contemporary examples of medical racism [43]. We found that linguistic isolation was more prevalent in highly positive census tracts. Though Public Health–Seattle & King County publishes information on open access testing in 14 different languages, community organizations have pointed out that immigrant communities often rely on word of mouth or social media for COVID-19 information and may miss updates from the public health agency [41]. Lack of time off work, fear that a positive result could lead to loss of income, and fear of authorities if undocumented have been reported as additional barriers to preventive testing in immigrant communities [44]. The fact that similar barriers are likely contributing to reported inequities in vaccination rates [45] underlines the importance of expanding trustworthy, culturally competent prevention resources for minoritized populations.

To our knowledge, this is the first study to assess the relationship between COVID-19 and environmental exposures in King County. Using a logistic regression model that adjusted for potential environmental confounders and population density, we found that PM_2.5_ was positively associated with high community positivity, whereas NO_x_ concentration was negatively associated. Though most studies that have assessed links between NO_x_ and COVID-19 incidence have found a significant positive correlation [46], in Milan, Italy, Zoran et al. detected an inverse relationship between NO_2_ and rates of COVID-19 infection [47]. Their explanation, that lower NO_x_ emissions lead to increased ozone concentration which may negatively impact respiratory health and thus COVID susceptibility, is possible in King County (census tract–level NO_x_ and ozone indicators were negatively correlated; r = − 0.487). However, our study did not identify a significant association between ozone and high COVID-19 positivity after adjustment for NO_x_, PM_2.5_, traffic density, and toxic waste emissions. Our finding that highly positive census tracts faced significantly higher levels of PM_2.5_ even after adjustment for population density adds to the growing body of international evidence that a correlation exists between PM_2.5_ and COVID-19 [23–25]. Particulate matter may be involved in the direct transmission of COVID-19. Setti et al. showed that PM is a carrier of SARS-CoV-2 in northern Italy, indicated by the presence of viral RNA [48]; Martelletti et Martellitti [49] argue that the virus may be absorbed onto PM, thus surviving longer and becoming more aggressive in the immune system; emphasizing the value of considering air quality in addition to social determinants of health when accounting for racial disparities in COVID-19. PM_2.5_ has been widely associated with respiratory conditions like asthma, bronchitis, pneumonia, and chronic obstructive pulmonary disease (COPD), all of which increase the likelihood of severe COVID-19 [22]. Though daily concentrations of PM_2.5_ varied by only 0.87 μg/m^3^ between King County positivity groups, a nationwide study of more than 3000 US counties found that an increase of only 1 μg/m^3^ in PM_2.5_ was associated with an 8% increase in the COVID-19 death rate [50]. The magnitude of wildfires in Washington State in 2020 may also have amplified the impact of PM_2.5_ on the region’s COVID-19 outbreak [51]. Disproportionate exposure to air pollution thus adds to the cumulative burden borne by minoritized, low-income, health-compromised communities in south King County. However, large numbers of essential workers and residents who use public transport may also be contributing to high positivity in areas with greater concentrations of PM_2.5_.

In addition to social, health-related, and environmental drivers of community COVID-19 positivity, other underlying factors may be contributing to a high proportion of tests coming back positive. Lower testing rates brought on by limited access to and/or use of testing may have contributed to high positivity virally, by allowing cases to circulate undetected in the community, but also mathematically, by decreasing the size of the denominator. Odds ratios from univariable regression analysis suggest a larger relative importance of positive case rates in driving high positivity, emphasizing heightened vulnerability to community spread. Nevertheless, a tendency for symptomatic people who are more likely to be positive for COVID-19 to seek testing, and the fact that testing is done on a voluntary basis, may be contributing to positive case rates and population characteristics that are not entirely representative of the underlying community.

Given our findings that highly COVID-19-positive communities face cumulative risks to environmental and social inequities, community-level investments for mitigating cumulative environmental health impacts are of critical importance. Community-based participatory research (CBPR), for example, could be a powerful tool for identifying tailored intervention strategies and policy change to reduce COVID-19 disparities in communities facing disproportionate cumulative impacts. Our study points to a need for increased understanding of how identified social inequities are contributing to racial disparities in COVID-19; of how to improve social and healthcare services and COVID-19 prevention and control resources for vulnerable communities; and of the mechanisms by which environmental exposures are exacerbating COVID-19 disparities in King County. CPBR partnerships, by engaging researchers and community stakeholders as equal partners in the research process, would help prioritize these research questions, translate findings into policy, and invest in highly impacted communities by helping residents to acquire new skills and become community leaders [52].

To address underlying socioeconomic inequities contributing to COVID-19 disparities, research suggests that accessible high-quality education programs, housing quality and mobility programs, income supplements, and employment interventions for specific vulnerable groups could all be effective interventions for reducing health disparities [50]. To address community health inequities, efforts must be made to support community-based health clinics and organizations that provide essential primary care services to underserved communities. The work of many of these clinics, including home visitations and other community outreach programs, has been curtailed rather than upscaled due to the COVID-19 pandemic [53]. Research also suggests that policies and programs that increase awareness of and access to healthy foods, when accompanied by skill-building programs to improve food shopping and consumption behaviors, can reduce BMI-related health inequities [54]. To reduce environmental inequities in highly impacted communities, our study results suggest that federal and statewide air pollution reduction initiatives and increased PM_2.5_ filtration materials in the home and workplace, as recommended by Huang et al. [55], may be important places to start.

In the introduction, we argued that a cumulative impact approach can provide a novel framework for more equitable allocation of resources. Indeed, we found that COVID-19 risk shared a great deal of spatial overlap with other environmental risks which was neither obvious nor quantified before our analysis. From an equity standpoint, policies and resources directed towards COVID-19 prevention and control should acknowledge and address these overlapping risks. COVID-19 prevention and control resources could be used to improve indoor air quality; environmental policies and resources could be used to improve infectious disease preparedness and mitigation.

### Limits

Our assessment of cumulative impacts in highly positive census tracts was limited by several factors. Because American Indian, Alaska Native, Native Hawaiian, and other Pacific Islander communities make up only 1.3% of King County’s total population, community-level analysis provided limited understanding of how COVID-19 and underlying disparities were affecting these populations specifically. Though analyzing cumulative COVID-19 rates allowed us to assess long-term disparities, lack of data on daily/weekly testing and case rates prevented us from assessing the role of temporal dynamics of the epidemic on COVID-19 outcomes. Data on respiratory health outcomes like COPD and asthma were not publicly available at the census tract level and associations between respiratory health and COVID-19 positivity could not be examined. Finally, it is important to note the potential limitations of using census tracts as units for evaluating community-level drivers of disease burden. Analyzing the residential locations of people testing positive for COVID-19 and seeking testing, while the finest resolution analysis possible with publicly available data, may not account for the spatial extent of individuals’ activities relating to COVID-19 risk (individuals may work and/or commute to other neighborhoods, or even counties). Unfortunately, data on essential jobs were unavailable at the census tract level and we could not account for geographic spillover of work-related risk as part of our analysis.

### Public Health Implications

This study highlights the extent to which communities with high COVID-19 positivity in King County face cumulative risks to environmental and social inequities. This suggests that cumulative environmental health impacts should be systematically considered when assessing risk of exposure to and health complications resulting from COVID-19. Our findings also indicate that applying existing environmental health mapping tools to the COVID-19 response and at the outset of future epidemics could be a rapid and effective way to identify the potential for, and prevent, health disparities in vulnerable communities by guiding prioritization of community interventions and investments to protect disproportionately exposed populations.

Our findings underline the continued need for disease prevention and control resources in the southwestern region of King County. Government and academic institutions should work closely with community-based organizations like cultural centers, faith-based, and community organizations on strategies for improving preventive services access and use and decreasing positivity rates in minoritized communities. These strategies should focus on the unique strengths in these communities that can help to combat and prevent COVID-19. From a structural standpoint, long-term interventions focusing on economic empowerment, community health programs, and clean air initiatives are important considerations for addressing root causes of community vulnerability to COVID-19. Future research is needed on how respiratory health outcomes and risk of exposure through essential jobs fit into the COVID-19 cumulative impacts framework, and on how the COVID-19 pandemic is affecting underrepresented populations like American Indians, Alaska Natives, Native Hawaiians, and other Pacific Islanders. With widespread COVID-19 vaccination programs underway, it is critical that cumulative impacts assessment be effectively applied to prioritize those most in need of protection.

## Supplementary Information

Supplementary Figure 1 (JPG 542 kb)
