# Supplementary figures and images for: Cumulative Impacts and COVID-19: Implications for Low-Income, Minoritized, and Health-Compromised Communities in King County, WA
Source: J Racial Ethn Health Disparities. 2021 Jun 14;9(4):1210–24. doi: 10.1007/s40615-021-01063-y (PMC8202963; doi:10.1007/s40615-021-01063-y)

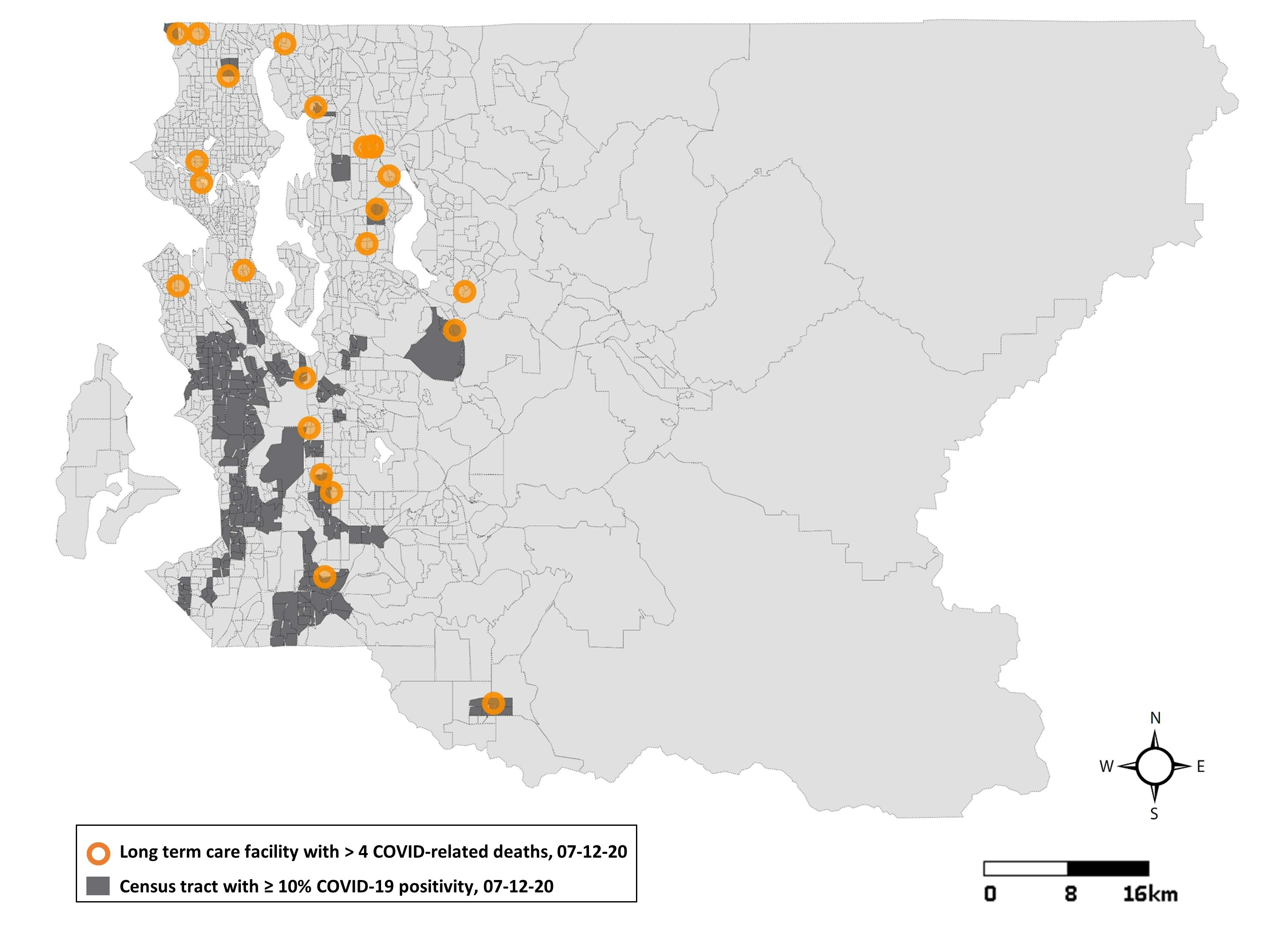

Supplement: Supplementary file 1 — (JPG 542 kb) [file 40615_2021_1063_MOESM1_ESM.jpg]
